# Cdk8 and Ssn801 Regulate Oxidative Stress Resistance and Virulence in Cryptococcus neoformans

**DOI:** 10.1128/mBio.02818-18

**Published:** 2019-02-12

**Authors:** Andrew L. Chang, Yiming Kang, Tamara L. Doering

**Affiliations:** a Department of Molecular Microbiology, Washington University School of Medicine, Washington University, St. Louis, Missouri, USA; b Department of Computer Science and Engineering, Washington University, St. Louis, Missouri, USA; Duke University Medical Center; Geisel School of Medicine at Dartmouth; University of British Columbia

**Keywords:** Cdk8, *Cryptococcus neoformans*, Mediator complex, Ssn801, mitochondria

## Abstract

Cryptococcus neoformans kills 200,000 people worldwide each year. After inhalation, this environmental yeast proliferates either extracellularly or within host macrophages. Under conditions of immunocompromise, cryptococci disseminate from the lungs to the brain, causing a deadly meningoencephalitis that is difficult and expensive to treat. Cryptococcal adaptation to the harsh lung environment is a critical first step in its pathogenesis, and consequently a compelling topic of study. This adaptation is mediated by a complex transcriptional program that integrates cellular responses to environmental stimuli. Although several key regulators in this process have been examined, one that remains understudied in C. neoformans is the Mediator complex. In other organisms, this complex promotes transcription of specific genes by increasing assembly of the RNA polymerase II preinitiation complex. We focused on the Kinase Module of Mediator, which consists of cyclin C (Ssn801), cyclin-dependent kinase 8 (Cdk8), Med12, and Med13. This module provides important inhibitory control of Mediator complex assembly and activity. Using transcriptomics, we discovered that Cdk8 and Ssn801 together regulate cryptococcal functions such as the ability to grow on acetate and the response to oxidative stress, both of which were experimentally validated. Deletion of *CDK8* yielded altered mitochondrial morphology and the dysregulation of genes involved in oxidation-reduction processes. This strain exhibited increased susceptibility to oxidative stress, resulting in an inability of mutant cells to proliferate within phagocytes, decreased lung burdens, and attenuated virulence *in vivo*. These findings increase our understanding of cryptococcal adaptation to the host environment and its regulation of oxidative stress resistance and virulence.

## Abstract (precis)

Cryptococcus neoformans is a fungal pathogen that primarily affects severely immunocompromised patients, resulting in 200,000 deaths every year. This yeast occurs in the environment and can establish disease upon inhalation into the lungs of a mammalian host. In this harsh environment it must survive engulfment by host phagocytes, including the oxidative stresses it experiences inside them. To adapt to these challenging conditions, C. neoformans deploys a variety of regulatory proteins to alter gene expression levels and enhance its ability to survive. We have elucidated the role of a protein complex that regulates the cryptococcal response to oxidative stress, survival within phagocytes, and ability to cause disease. These findings are important because they advance our understanding of cryptococcal disease, which we hope will help in the efforts to control this devastating infection.

## INTRODUCTION

Cryptococcus neoformans is a fungal opportunistic pathogen that is found ubiquitously throughout the environment. Mammalian hosts inhale desiccated cryptococcal cells or spores into the lungs, where they interact with the innate immune system, frequently being engulfed by phagocytes. This facultative intracellular pathogen must thus be able to survive not only the hostile, low-nutrient pulmonary environment but also the stressful conditions within host phagocytes (1–3). If it is unable to adapt to these changes, it is cleared. Otherwise, it may remain in the lung in a latent state or, under conditions of immunocompromise, disseminate throughout the body (2, 4). This dissemination shows a tropism for the brain and causes a lethal meningoencephalitis that results in 200,000 deaths every year (5).

Cryptococcal adaptation to the intracellular and host environment is key to its ability to cause disease. This adaptation begins with transcriptional changes that regulate its ability to grow at mammalian host temperatures, to survive environmental stresses and limited resource environments, and to produce critical cryptococcal virulence factors. Although many transcriptional regulators have been well studied in C. neoformans (6–10), one important protein complex that modulates transcription has not been addressed: the Mediator complex. We identified Cdk8, a component of this complex, in a screen for cryptococcal genes that affect interactions with a macrophage cell line (11). This led to our efforts to determine its role in cryptococcal biology and virulence.

In Saccharomyces cerevisiae, Mediator is composed of 25 proteins, grouped in four modules, and assists in the assembly of the RNA polymerase II preinitiation complex (12–14). Three modules of Mediator comprise a base complex that interacts directly with RNA polymerase II to facilitate transcription (14). The last module of Mediator is the Kinase Module, which consists of four proteins: cyclin C (Ssn8), cyclin-dependent kinase 8 (Cdk8), Mediator subunit 12 (Med12), and Mediator subunit 13 (Med13) (14).

The components of the Kinase Module modify transcription through several well-understood mechanisms. In one, Cdk8 phosphorylates the carboxy-terminal domain of RNA polymerase II. This directly inhibits assembly of the preinitiation complex, thus repressing transcription (14). In another, Cdk8 and Ssn8 together allosterically inhibit the interaction of the Mediator base complex and RNA polymerase II, which in turn results in decreased assembly of the preinitiation complex that is required for transcription to proceed (14). These two mechanisms are also influenced by other Kinase Module components, with Med12 likely playing a larger role in the first, and Med13 in the second (15). Importantly, even though the Kinase Module does not appear to contain a DNA binding domain, there is still evidence that it influences specific targets rather than broadly repressing transcription (see Discussion). Another way for Cdk8 and Ssn801 to directly control mitochondrial morphology has been suggested based on observations in S. cerevisiae (16, 17). In this mechanism, Ssn8 first interacts with Cdk8. It then independently exits the nucleus to localize at the mitochondria, where it recruits proteins involved in mitochondrial fission to promote this process (16).

Experiments in several fungal systems highlight the importance of Cdk8 and Ssn801. For example, deletion of either gene in S. cerevisiae results in cells with decreased mitochondrial fission and increased resistance to oxidative stress (18). In Candida albicans, disruption of Cdk8 and other Mediator elements alters regulation of virulence in a murine infection model (19–22). Finally, studies in Candida glabrata have shown that Mediator influences the development of antifungal resistance (23).

We studied the roles of Cdk8 and Ssn801 in cryptococcal biology and virulence and found that this protein pair is critical in the transcriptional adaptation of C. neoformans to the host. Through analysis of the cryptococcal transcriptome using targeted mutant strains, we specifically implicated Cdk8 and Ssn801 in the regulation of two key processes, the glyoxylate cycle and the cryptococcal response to oxidative stress. The glyoxylate cycle allows organisms to survive on nonfermentable carbon sources; consistent with our analysis, mutants lacking any of the Kinase Module components were impaired in their ability to grow on acetate. Lack of Cdk8 and Ssn801 also caused intracellular survival defects contingent on oxidative stress susceptibility, alterations in mitochondrial morphology and function, and decreased virulence *in vivo*. These two proteins thus play critical roles in the ability of a dangerous pathogen to regulate its adaptation to the host environment and thereby cause disease.

## RESULTS

**FIG 1:**
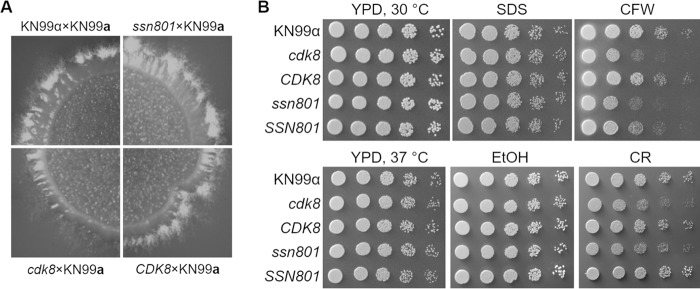
Cells lacking Cdk8 show reduced filamentation and resistance to cell wall stress. (A) Crosses of KN99**a** to the *cdk8* strain (bottom left) produce fewer mating filaments than crosses with the WT, *ssn801*, or complemented *CDK8* strain. Plates were imaged 10 days after crossing was initiated as in Materials and Methods. (B) The *cdk8* and *ssn801* strains show increased susceptibility to calcofluor white (CFW; 0.05% [wt/vol] at 37°C) and Congo red (CR; 0.005% at 30°C) but no growth defect in the presence of SDS (0.01% [wt/vol] sodium dodecyl sulfate, 30°C), elevated temperature alone (37°C), or 5% (vol/vol) ethanol (EtOH; the control for Congo red plates, 30°C). *CDK8*, *cdk8* strain complemented with the WT gene at the native site; *SSN801*, *ssn801* strain complemented with the WT gene at the native site.

Results from prior studies of the interaction between C. neoformans and macrophages suggested that the product of CNAG_06086 might influence this process (11). The first ortholog identified by BLASTp analysis of this sequence against the S. cerevisiae genome was a protein named Cdk8, which was 45% identical at the amino acid level to the predicted product of CNAG_06086. Using the S. cerevisiae Cdk8 primary sequence, we performed a second BLASTp analysis against the cryptococcal H99 genome and found that CNAG_06086 was the ortholog with the most significant E value (5 × 10^−126^). We therefore named CNAG_06086 *CDK8*, in accordance with nomenclature guidelines of the field (24). Cdk8 has a binding partner in S. cerevisiae named Ssn8. In C. neoformans, an ortholog of Ssn8 (encoded by CNAG_00440, *SSN801*) has been previously reported (25). These studies showed that *ssn801* deletion strains have a defect in mating, so we generated *cdk8* and *ssn801* deletions strains (see Materials and Methods) and assessed their mating. Interestingly, our *cdk8* mutant showed reduced production of mating filaments, although unlike the previous results, our *ssn801* mutant did not (Fig. 1A); this difference may be due to the use of different background strains (KN99 versus H99). The earlier studies also reported that the *ssn801* mutant had reduced resistance to Congo red, which induces cell wall stress. The *ssn801* and *cdk8* mutants that we generated similarly exhibited a defect when grown with the cell wall stressors Congo red and calcofluor white but not when grown in the presence of SDS or ethanol (Fig. 1B).

**FIG 2:**
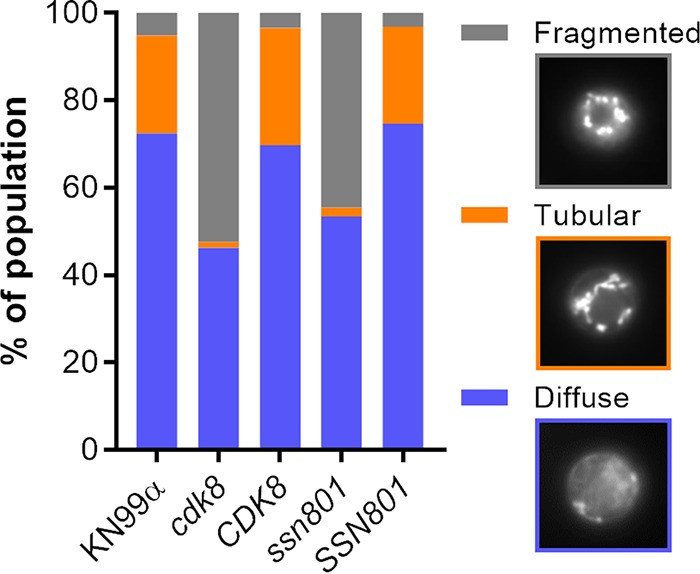
Deletion of *CDK8* or *SSN801* results in abnormal, fragmented mitochondrial morphology. Mitochondrial morphology assays of the parental WT strain KN99α, the *cdk8* strain, the complemented *CDK8* mutant, the *ssn801* strain, and the complemented *SSN801* mutant. Categories of morphology (examples shown at right) are indicated as follows: gray, fragmented; orange, tubular; blue, diffuse. At least 150 cells per sample were categorized in a double-blinded manner, and the results shown are representative of 3 experiments.

As mentioned above, in S. cerevisiae deletion of the genes encoding either Ssn8 or its binding partner Cdk8 results in decreased mitochondrial fission (yielding less fragmentation of mitochondria) and enhanced resistance to oxidative stress. To test whether mitochondrial morphology is similarly affected in C. neoformans, we grew our mutants in tissue culture medium, stained them with the mitochondrion-specific dye MitoTrackerCMXRos, and characterized their morphology in a blinded manner. Tissue culture medium was chosen as a “host-like” condition that resembles the context under which C. neoformans would be most likely to experience oxidative stress (26). Morphology was categorized as diffuse, where the mitochondria are distributed in a thin, reticular network without strong focal regions of fluorescence; fragmented, where the dominant impression is of individual puncta scattered throughout the cell; or tubular, where large fused tubules may be observed (see Fig. S1 in the supplemental material). In wild-type (WT) cells, most mitochondria were diffuse, with almost all of the rest adopting a tubular morphology (Fig. 2) (27). Surprisingly, the deletion of either gene resulted in markedly increased mitochondrial fragmentation, rather than the decreased fragmentation we expected based on S. cerevisiae. This effect was reversed by complementation with each WT gene (Fig. 2).

10.1128/mBio.02818-18.1 FIG S1 Additional examples of categories of mitochondrial morphology. Wild-type cells were grown and mitochondrial morphology assessed as in Materials and Methods. Example micrographs of the different categories of mitochondrial morphology are shown. Scale bar, 10 μm. The top and bottom panels in the Diffuse column are shown at slightly lower brightness for clarity, and the second panel in the Tubular column shows a larger cell exhibiting tubular morphology together with a smaller cell of fragmented morphology. Download FIG S1, PDF file, 0.1 MB. Copyright © 2019 Chang et al. 2019 Chang et al. This content is distributed under the terms of the Creative Commons Attribution 4.0 International license.

**FIG 3:**
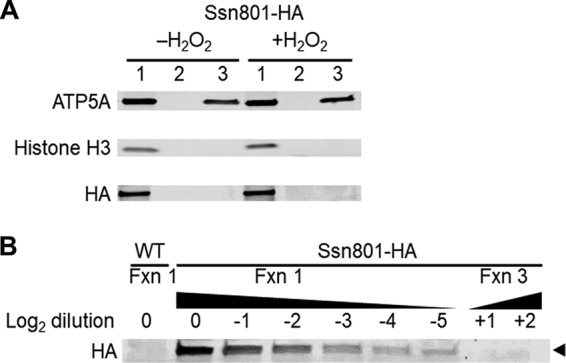
Localization of Ssn801-HA by immunoblotting. (A) Equal cell equivalents of various cell fractions (see Fig. S2A) were probed for markers of mitochondria (ATP5A) and nuclei (histone H3), as well as for Ssn801-HA. Lanes: 1, 1,500 × *g* pellet (nuclei and unbroken cells); 2, 4,000 × *g* pellet (organelles that sediment slower than nuclei but faster than mitochondria); 3, 15,000 × *g* pellet (mitochondria). Neither marker is present in fraction 2 (see also Fig. S2B). Ssn801-HA occurs only in the fraction containing nuclei, even when cells are treated with 1.5 mM H_2_O_2_ to induce stress. (B) Samples normalized as in panel A were serially diluted (fraction 1) or loaded in greater amounts (fraction 3) to demonstrate the limit of detection. No Ssn801 is detected in the mitochondrial fraction even at 128-fold-higher cell equivalents than the nuclear fraction. As expected, it is also absent from untagged cells (WT).

We wondered whether the unexpected, reversed change in mitochondrial morphology was still mediated by direct interactions of Ssn801 with mitochondrial proteins. To probe such interactions, we generated a strain expressing a C-terminally HA-tagged version of Ssn801, which functioned like the WT protein (see below). When we used this strain for proteomic analysis of coprecipitated proteins, however, we detected no specific interactions with mitochondrial proteins, despite multiple experiments. This result raised the question of whether Ssn801 translocates from the nucleus to mitochondria in C. neoformans as it has been reported to do in S. cerevisiae. To test this, we developed a fractionation method to reliably separate mitochondrial from nuclear markers (Fig. S2) and applied it to cells expressing Ssn801-HA. In these studies, we detected abundant Ssn801-HA in the fraction containing nuclei, but none in the mitochondrial fraction (Fig. 3A), even though our method is sensitive enough to detect Ssn801-HA even if significantly less than 1% of the total protein has translocated (Fig. 3B). Together, these results strongly suggest that C. neoformans has no process analogous to the direct action of Ssn8 at mitochondria in S. cerevisiae.

10.1128/mBio.02818-18.2 FIG S2 Subcellular fractionation of C. neoformans. (A) Schematic of isolation of mitochondria by subcellular fractionation; see Materials and Methods for details. (B) Immunoblot of fractions indicated in panel A. Nuclei, marked by histone H3 (16 kDa), are present only in the total lysate (TL) and 1,500 × *g* pellet (1). In contrast mitochondria, marked by ATP5A (55 kDa), are present in the TL, 1,500 × *g* pellet (1; in unbroken cells), and the 15,000 × *g* pellet (3). The band at ∼29 kDa is a consistent background band from the anti-ATP5A antibody. L, ladder. Download FIG S2, PDF file, 0.1 MB. Copyright © 2019 Chang et al. 2019 Chang et al. This content is distributed under the terms of the Creative Commons Attribution 4.0 International license.

Although Ssn801 does not appear to directly act at mitochondria, it is clear that both it and Cdk8 markedly influence cryptococcal mitochondrial morphology (Fig. 2). We suspected this might occur via one of the transcriptional mechanisms discussed above and chose a transcriptomic approach to test this hypothesis. In addition to the *cdk8* and *ssn801* deletion strains already in hand for these studies, we engineered a strain expressing a version of Cdk8 lacking kinase activity (kinase-dead Cdk8 [Cdk8^KD^]; see Materials and Methods), as well as a parallel control strain bearing the WT version of Cdk8 (Cdk8^WT^). We also deleted the genes encoding the remaining two components of the Kinase Module, Mediator subunits 12 and 13 (*MED12* and *MED13,* respectively).

**FIG 4:**
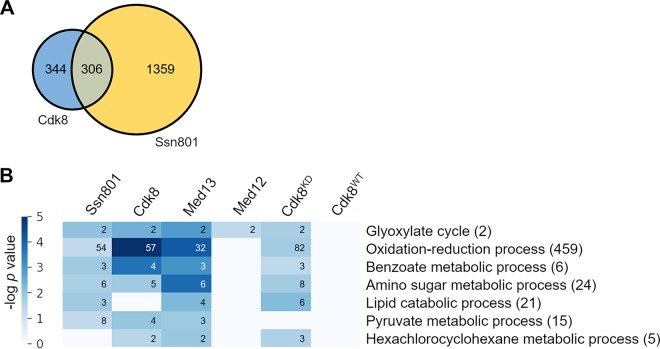
RNA-seq analysis of the transcriptional effects of Cdk8 and Ssn801 deletion. (A) The number of DE genes from RNA-seq analysis of *cdk8* and *ssn801* strains and the overlap of these sets. (B) RNA-seq of experimental strains was analyzed for GO categories that were enriched in DE genes upon comparison to WT. First five columns: categories enriched for genes with decreased transcription upon lack of the indicated proteins or of Cdk8 kinase activity, i.e., genes that are normally activated by these proteins or Cdk8 kinase activity. Sixth column: Cdk8^WT^ control. Activated GO term categories are listed at the right. The numbers following each GO term indicate the number of genes in that category; the number of these that are significantly altered in each strain is shown in the columns. The colored scale indicates the statistical significance of enrichment from 10^0^ to 10^−5^ (most significant).

We performed RNA-seq on the KN99α, *cdk8*, *ssn801*, *med12*, *med13*, Cdk8^KD^, and Cdk8^WT^ strains and identified differentially expressed (DE) genes, which we defined as those with an absolute fold change in expression of at least 2-fold with a multiple-hypothesis adjusted *P* value of <0.01 (see Materials and Methods and Table S1). When we compared the DE lists for the *cdk8* and *ssn801* mutant strains, we found that almost half of the genes regulated by Cdk8 were also regulated by Ssn801 (Fig. 4A). This overlap greatly exceeds that predicted by random chance (*P* value < 10^−12^ in a hypergeometric test) and suggests that these proteins act together to regulate transcription.

10.1128/mBio.02818-18.8 TABLE S1 Differentially expressed genes from RNA-seq analysis. Differentially expressed genes obtained from RNA-seq analysis. Individual spreadsheets list genes that were upregulated (activated) or downregulated (repressed) in each engineered strain compared to WT, along with the fold change and adjusted *P* value. Download Table S1, XLSX file, 0.4 MB. Copyright © 2019 Chang et al. 2019 Chang et al. This content is distributed under the terms of the Creative Commons Attribution 4.0 International license.

Our RNA-seq studies showed that Cdk8 and Ssn801 regulate the expression of multiple genes, which may in turn regulate various aspects of cellular function, either directly or indirectly. To relate this regulatory activity to mitochondrial function, we performed gene set enrichment analysis of the DE gene lists from each mutant strain. After categorizing each DE cryptococcal gene into a gene ontology (GO) term or category (Table S2), we assessed which categories were enriched in each engineered strain compared to KN99α. GO categories that were repressed upon deletion of a gene (or interference with Cdk8 kinase activity) were considered to be activated by that gene (or kinase activity) (Table S3). The top activated GO terms, selected as described in Materials and Methods, are shown in Fig. 4B.

10.1128/mBio.02818-18.9 TABLE S2 Cryptococcal genes assigned to each of the top 10 identified GO terms. Individual spreadsheets for each of the top 10 identified GO terms list genes that were differentially expressed for each engineered strain (noted on the top line). These data were used to build Fig. 4. Download Table S2, XLSX file, 0.03 MB. Copyright © 2019 Chang et al. 2019 Chang et al. This content is distributed under the terms of the Creative Commons Attribution 4.0 International license.

10.1128/mBio.02818-18.10 TABLE S3 Enriched activated and repressed GO terms. GO categories that exhibited statistically significant activation or repression of gene expression in at least one strain tested are listed, along with the number of genes affected and the total number of genes in the category. Download Table S3, XLSX file, 0.02 MB. Copyright © 2019 Chang et al. 2019 Chang et al. This content is distributed under the terms of the Creative Commons Attribution 4.0 International license.

**FIG 5:**
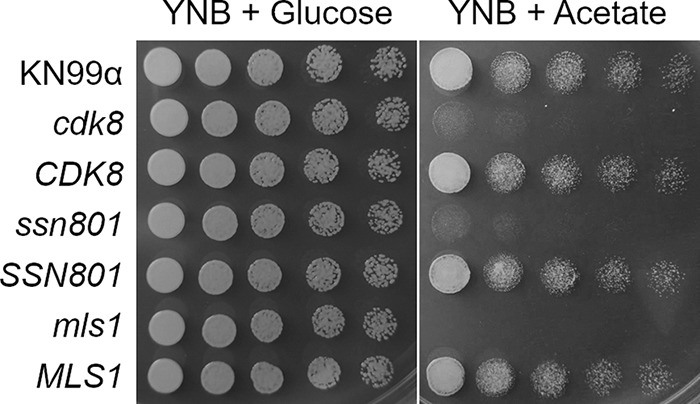
Cells lacking Cdk8 and Ssn801 are impaired in growth on acetate. Serial dilutions of the strains listed at the left were grown on YNB containing glucose or acetate as the carbon source (see Materials and Methods for details).

One gene set predicted to be regulated by the Cdk8/Ssn801 system encodes the components of the glyoxylate pathway; this controls growth on simple carbon sources when complex carbon sources are absent by enabling the conversion of acetyl-CoA to succinate to generate carbohydrates (28, 29). There are two key proteins in this process: malate synthase (Mls1) and isocitrate lyase (Icl1). The expression of both corresponding genes was strongly repressed in the *cdk8*, *ssn801*, *med13*, *med12*, and Cdk8^KD^ mutant strains, but not in the Cdk8^WT^ control strain. To test whether this reduced expression was manifested as dysregulation of the glyoxylate cycle in these strains, we measured the ability of these strains to grow using sodium acetate as a carbon source. All of them showed reduced growth under these conditions compared to their matched controls, with the impairment comparable to that seen upon complete deletion of *MLS1* (Fig. 5 and Fig. S3, top two panels). This confirmed our expectations based on the RNA-seq results.

10.1128/mBio.02818-18.3 FIG S3 Growth of mutant strains on acetate and under oxidative stress with sorbitol. Serial 10-fold dilutions (10^7^ to 10^4^ cells/ml) of the strains listed at the left were tested for glyoxylate cycle function by growth on YNB with glucose or acetate (top two panels) or were tested for growth under peroxide stress with or without sorbitol (bottom panel). Download FIG S3, PDF file, 0.1 MB. Copyright © 2019 Chang et al. 2019 Chang et al. This content is distributed under the terms of the Creative Commons Attribution 4.0 International license.

Having biologically validated one process suggested by GO annotation of our RNA-seq results, we examined these annotations for other processes that might influence fungal physiology and potentially virulence. One category of interest was genes involved in oxidation-reduction processes; this was dysregulated in all strains tested except for the control Cdk8^WT^ strain. This gene set was particularly interesting, not only because it was the second most significantly dysregulated pathway in the experimental strains but because this process is crucial to survival of fungi within the intracellular environment. Furthermore, deletion of *CDK8* and *SSN801* homologs in S. cerevisiae increases resistance to oxidative stress. To determine whether the GO/KEGG analysis correctly predicted altered susceptibility to oxidative stress, we focused on the *cdk8* and *ssn801* deletion mutants.

**FIG 6:**
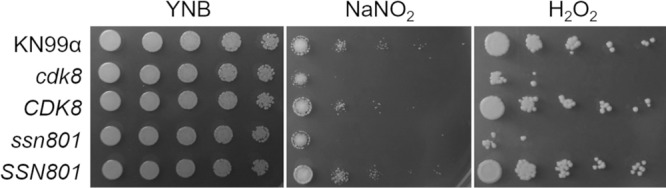
Cells lacking Cdk8 and Ssn801 have increased sensitivity to nitrosative and oxidative stress. Serial dilutions of the indicated strains were grown either on YNB alone or YNB containing either NaNO_2_ or H_2_O_2_ at 1 mM (see Materials and Methods for details).

We first tested the *cdk8* and *ssn801* mutant strains for their ability to grow in the presence of 1 mM NaNO_2_ and H_2_O_2_ to impose nitrosative and oxidative stresses. In surprising contrast to S. cerevisiae, the mutant strains showed a significant decrease in resistance to both stresses compared to their complements and WT (Fig. 6). We considered the possibility that this phenotype was related to the cell wall defects we had observed earlier, which may influence the ability of these stressors to enter the cell. To test this, we added 1 M sorbitol to the oxidative stress medium to provide osmotic support, but this did not rescue mutant growth (Fig. S3, bottom panel).

**FIG 7:**
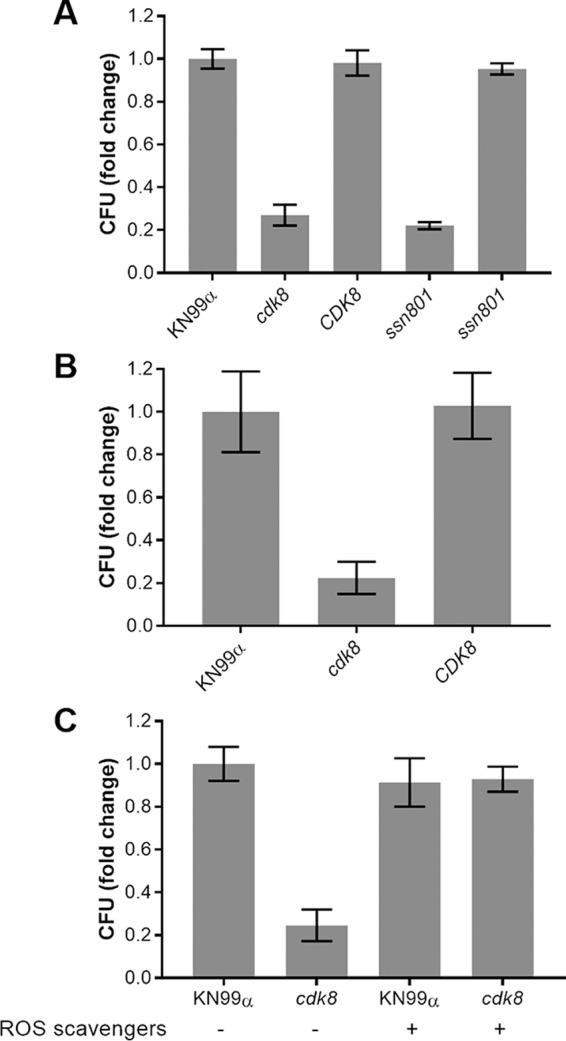
Defects in intracellular survival are reversed by ROS scavengers. Intracellular survival (mean ± SD) of the indicated strains is shown as fold change, normalized to WT (typically 1.5- to 2.0-fold change). Where *P* value is not indicated below, there was no significant difference from WT. (A) 48-h survival in THP-1 cells. *P *≤* *0.0001 for *cdk8* and *ssn801* strains compared to WT. (B) 24-h survival in BMDM. *P* ≤ 0.001 for *cdk8* strain compared to WT. (C) 24-h survival in THP-1 cells, in the absence and presence of ROS scavengers. *P* ≤ 0.0001 for *cdk8* strain compared to WT. Panels A and B are representative of three independent experiments, and panel C is representative of two independent experiments.

We speculated that susceptibility to oxidative and nitrosative stress would compromise the survival of *cdk8* and *ssn801* cells within phagocytes. Indeed, despite similar levels of phagocytosis, both mutants showed clear and reproducible defects in intracellular survival in differentiated THP-1 cells (an immortalized human monocyte line [Fig. 7A]). These defects were reversed in the corresponding complemented strains; survival of these and of the Ssn801-HA strain was like that of WT cells (Fig. S4). Reduced virulence of an *ssn801* strain has previously been reported, so we focused on our *cdk8* strain. We next tested its intracellular survival in primary bone marrow-derived macrophages (BMDM), rather than a cell line. Consistent with the prior THP-1 results, the *cdk8* cells showed a significant defect in survival in primary bone marrow-derived macrophages (BMDM, Fig. 7B), despite WT growth rates in the RPMI medium used for this study (Fig. S5).

10.1128/mBio.02818-18.4 FIG S4 Ssn801-HA strains show WT levels of survival in THP-1 macrophages compared to the *ssn801* mutant strain. Intracellular survival (mean ± SD) of the indicated strains is shown as fold change, normalized to WT (1.8-fold change). Results shown are representative of three independent experiments. *P* ≤ 0.0001 for *ssn801* strain compared to WT. Download FIG S4, PDF file, 0.1 MB. Copyright © 2019 Chang et al. 2019 Chang et al. This content is distributed under the terms of the Creative Commons Attribution 4.0 International license.

10.1128/mBio.02818-18.5 FIG S5 *cdk8* cells grow like WT in YPD and RPMI (although not DMEM) and exhibit normal capsule. Top row, growth curves (mean ± SD) of WT (KN99α, blue), *cdk8* (orange), and complemented mutant (*CDK8*, gray) strains in YPD (A), RPMI (B), and DMEM (C). RPMI medium was used in the studies reported in the intracellular survival studies in the main text. Bottom left, example micrographs of the indicated strains after 24 h of incubation under capsule-inducing conditions (DMEM, 37°C, 5% CO_2_) and India ink staining. All images are to the same scale; scale bar, 10 µm. Bottom right, capsule width measured for at least 100 cells chosen at random. Mean ± SD is plotted. *P* ≥ 0.05 for *cdk8* strain compared to WT. Download FIG S5, PDF file, 0.1 MB. Copyright © 2019 Chang et al. 2019 Chang et al. This content is distributed under the terms of the Creative Commons Attribution 4.0 International license.

We speculated that the reduced intracellular survival of *cdk8* cells was due to the marked susceptibility to oxidative stress that we had observed in these cells (Fig. 6), consistent with the perturbations suggested by our RNA-seq studies. To test whether reducing this stress would improve cell survival, we assessed intracellular survival in the absence and presence of a reactive oxygen species (ROS) scavenger cocktail. The application of ROS scavengers was sufficient to completely restore the ability of *cdk8* to proliferate within THP-1 macrophages (Fig. 7C), confirming our hypothesis.

**FIG 8:**
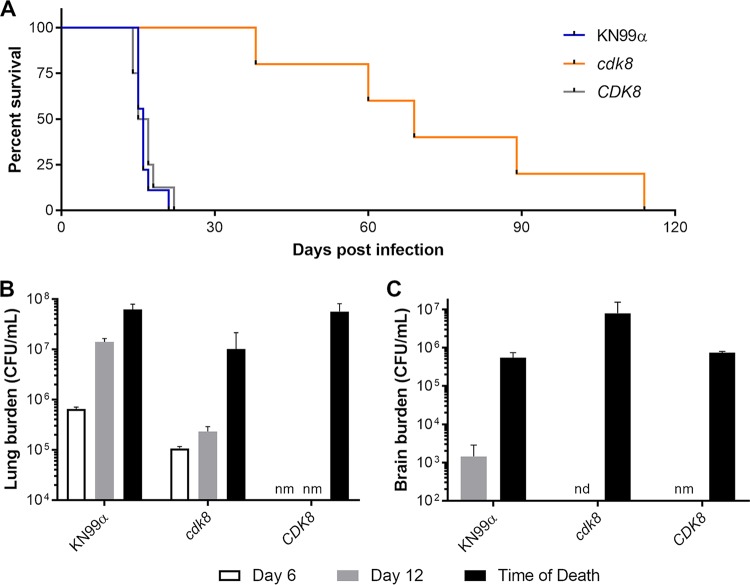
Mice infected with *cdk8* strain survive longer and have altered organ burden compared to WT-infected mice. (A) Survival curve for mice intranasally inoculated with 5 × 10^4^ cells of the indicated strains. (B and C) Lung (B) and brain (C) burdens of mice infected with the indicated strains for the times shown. nm, not measured; nd, none detected. Mean ± SEM is plotted.

The defects in intracellular proliferation of the *cdk8* mutant *in vitro* suggested that it would also be impaired in growth in its ability to cause disease in mice. Indeed, we found that while mice infected with WT or *CDK8* cells lost weight to the point of sacrifice in 3 weeks, those infected with the *cdk8* mutant showed a protracted survival curve (Fig. 8A). In parallel with the survival study, we plated lung and brain homogenates to determine organ burden at day 6 and day 12 of infection. The lung burdens of *cdk8* mutant-infected mice were low compared to those of WT-infected mice at both time points (Fig. 8B); this difference was most dramatic at day 12 (gray bars), near the onset of decline for the control mice. At the time of death for each mouse in the survival study (plotted in Fig. 8A), we also measured lung burden. This value (black bars) was also lower for mice infected with *cdk8* than for those infected with WT, a defect that was corrected in the complemented (*CDK8*) strain (assessed only at this time point) (Fig. 8B).

We wondered whether a deficiency in any known cryptococcal virulence traits contributed to the attenuated virulence of the *cdk8* strain. The best-known C. neoformans virulence factor is production of a large, polysaccharide capsule that is antiphagocytic and immunosuppressive. However, when we incubated *cdk8* cells under capsule-inducing conditions, we observed normal development of capsule (Fig. S5). Another well-studied cryptococcal virulence trait is the ability to produce melanin, which is involved in oxidative stress resistance. The *cdk8* strain showed a mild defect in its ability to produce melanin at higher temperatures (Fig. S6), which may contribute to its reduced virulence (see Discussion).

10.1128/mBio.02818-18.6 FIG S6 Cdk8 kinase activity is not required for melanin production but is required for survival on a cell wall stressor. (A) Melanization of Kinase Module mutants. The indicated strains (each 10^4^ cells) were grown at 30 and 37°C on l-DOPA agar to assess melanin production (see Materials and Methods for details). (B and C) Cdk8 kinase activity is required for robust growth in the presence of a cell wall stressor (B) and THP-1 macrophages (C). Serial 10-fold dilutions (10^7^ to 10^4^ cells/ml) of the strains listed at the left were grown on the medium indicated. Download FIG S6, PDF file, 0.1 MB. Copyright © 2019 Chang et al. 2019 Chang et al. This content is distributed under the terms of the Creative Commons Attribution 4.0 International license.

**FIG 9:**
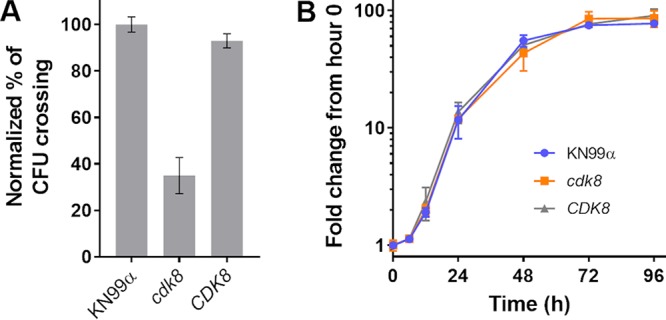
The *cdk8* mutant is impaired in blood-brain barrier crossing but grows normally in artificial CSF. (A) Efficiency of model BBB crossing (mean ± SD) for the indicated strains; results are representative of three independent experiments. *P* ≤ 0.0001 for *cdk8* strain compared to WT. (B) Growth curve for indicated strains in artificial CSF. Mean ± SD is plotted, and results are representative of two independent experiments.

Consistent with the lung burden results, we observed that the brain burden at day 12 for mice infected by the *cdk8* strain was significantly lower than that of mice infected by the WT strain (Fig. 8C) (nd, none detected). We were therefore surprised to find that mice infected by the mutant strain exhibited significantly higher brain burdens at the time of death than mice infected by the WT strain (Fig. 8C). One possible reason for this finding is that the *cdk8* strain, while unable to accumulate at normal rates in the lung, is exceptional at escaping the lung and crossing the blood-brain barrier (BBB). However, when we performed *in vitro* assays to measure the ability of these cells to cross an artificial BBB, we found that the *cdk8* strain was markedly impaired in this ability (Fig. 9A). An alternative explanation for the increased brain burden is that the *cdk8* strain grows more quickly within cerebrospinal fluid (CSF). To test this, we performed *in vitro* growth curves in artificial CSF but found no difference between growth of *cdk8* and control strains (Fig. 9B) (30).

## DISCUSSION

Cryptococcal adaptation to the host environment is critical for its ability to cause disease, and this adaptation is controlled by its transcription factors. These proteins control critical aspects of the transition to the host environment, such as adapting to alkaline pH and micronutrient deprivation (31, 32), producing capsule (10, 31), mating (10, 25), resisting cell wall stress (33), and resisting oxidative stress (34, 35). We have directly studied two transcriptional regulators, Cdk8 and Ssn801, and have demonstrated that these proteins regulate the cryptococcal response to oxidative stress.

Cdk8 and Ssn801 are components of the Mediator complex that regulates RNA polymerase II transcription, acting to modulate transcription via several independent mechanisms. Interestingly, this pair of proteins has also been implicated in a distinct regulatory pathway in S. cerevisiae, which relies on protein-protein interactions and the export of Ssn801 from the nucleus to directly modulate mitochondrial morphology (16, 17). We have observed striking differences in the function of this protein pair between C. neoformans and this model yeast. First, although C. neoformans Cdk8 and Ssn801 indeed modulate mitochondrial morphology, mitochondria in the corresponding mutants became more fragmented, instead of less fragmented. Second, the cryptococcal mutants show increased susceptibility to oxidative stress, also the opposite of what is seen in S. cerevisiae; this is consistent with, and may be due to, the altered mitochondrial morphology (27). Finally, we found no evidence that Ssn801 translocates to the mitochondria in C. neoformans. While we cannot rule out that this occurs in other conditions, the ones we tested are physiologically relevant to infection, and reflect the context in which oxidative stress is likely to be experienced by this pathogen.

In addition to altered mitochondrial morphology, deletion of *CDK8* and *SSN801* yielded mutants that were strongly attenuated in oxidative stress resistance, intracellular proliferation, and virulence. To probe these and other cellular phenotypes, we performed transcriptional profiling experiments. These studies indicated that the two proteins act together; that they influence multiple genes whose products act in varied cellular processes; and that they exhibit specificity, even though the lack of known DNA-binding domains might suggest that absence of these regulators would broadly decrease transcription. The last finding is consistent with specific dysregulation that has been observed when Cdk8 activity is impaired in other organisms. For example, microarray analysis comparing WT and kinase-dead Cdk8 in S. cerevisiae identified a set of only 173 genes with altered regulation, about 2.8% of total genes (15). In a human cell line, knockdown of *CDK8* expression resulted in 87 dysregulated genes (0.5%) (36). In C. neoformans, this system controls more genes: upon deletion of *CDK8* or *SSN801*, 8.1% and 20.7% of cryptococcal genes were dysregulated, respectively. We cannot explain why Ssn801 influences the expression of more genes; it may be that this protein has additional biological roles in C. neoformans that remain to be discovered.

The function of the Kinase Module suggests that it would be involved in inhibition of transcription, which we saw evidenced by the upregulation of 351 and 975 genes in *cdk8* and *ssn801* mutant cells, respectively. We were interested that our RNA-seq data also indicated gene activation (of 299 and 690 genes in *cdk8* and *ssn801* mutant cells, respectively). This may be due to the repression of genes whose products are negative regulators.

Deleting any of the four subunits of the Kinase Module (Cdk8, Ssn8, Med12, or Med13) in S. cerevisiae yields “virtually identical” gene expression profiles (37). Interestingly, in our studies the lack of Med12 impacts a set of GO terms that is distinct from those influenced in the other perturbed strains (Fig. 4B and Table S3). Furthermore, *med12* mutants grow slowly compared to the others, and are more sensitive to some stress conditions (e.g., Congo red, high temperature [Fig. S7]). This suggests that Med12 has additional roles in cryptococcal biology, potentially independent of Mediator components.

10.1128/mBio.02818-18.7 FIG S7 Cells lacking Med12, but not Med13, are defective in growth at 37°C and under some stress conditions. The strains indicated were plated on YPD medium under the conditions shown. CFW, 0.05% (wt/vol) calcofluor white at 37°C; SDS, 0.01% (wt/vol) sodium dodecyl sulfate at 30°C; 5% (vol/vol) ethanol at 30°C; CR, 0.005% Congo red and 5% (vol/vol) ethanol at 30°C. Download FIG S7, PDF file, 0.1 MB. Copyright © 2019 Chang et al. 2019 Chang et al. This content is distributed under the terms of the Creative Commons Attribution 4.0 International license.

Although the gene expression profiles of cells lacking Cdk8 and Ssn801are generally similar, they still hint at the possibility of differentiating between targets that are influenced by both proteins and those that are regulated by either one; they further suggest that some Cdk8 targets are regulated independently of kinase activity. This is supported by our phenotypic findings. For example, melanin production was slightly inhibited at host temperatures in the *cdk8* mutant, but not in the *ssn801* or Cdk8^KD^ mutant (Fig. S6A). This suggests that Cdk8 acts independently to regulate this important virulence trait and that this regulation is independent of its kinase activity. In contrast, in tests of cell sensitivity to CFW (Fig. S6B) or intracellular survival (Fig. S6C), mutating the kinase active site was enough to recapitulate the *cdk8* mutant phenotype; in these cases, therefore, the Cdk8 regulation of these phenotypes is kinase dependent. We have examined and validated several biological processes suggested by gene expression as targets of Cdk8. It would be interesting to examine additional biological pathways implicated by the GO category analysis, including metabolic pathways; recent studies in Candida albicans suggest this system regulates metabolism and biofilm formation (38). Clearly, more effort will be required to dissect the complex regulation mediated by this important pair of proteins and mechanistically define specific biochemical targets.

We observed several defects in the *cdk8* mutant that may contribute to its dramatically attenuated virulence, including altered mitochondrial morphology and increased susceptibility to some cell wall stressors as well as oxidative and nitrosative stress. All of these likely contribute to decreased intracellular survival and virulence, and some of these phenotypes may be related. For example, perturbed mitochondrial function may decrease resistance to oxidative stress (27) and influence cell wall biogenesis (39); cell wall defects in turn may influence interactions with host cells and stimulation of host responses. The *cdk8* mutant also exhibits decreased growth on the nonfermentable carbon source acetate, but this is unlikely to explain the strain’s overall decreased virulence given previous findings that glyoxylate cycle deletion mutants exhibit WT virulence (29).

The pattern of virulence of the *cdk8* mutant is striking, because decreased lung burdens are counterintuitively accompanied by increased brain burdens at the time of death. This result cannot be explained by increased blood-brain barrier crossing (the mutant crosses worse than WT) or by faster growth in CSF (the mutant matches WT proliferation in artificial CSF), although these conclusions are based on *in vitro* assays. One possible model, which accounts for all of our findings, begins with the decreased lung burden of *cdk8* cells, a result of their poor survival when confronted with lung phagocytes (Fig. 7). This lower burden results in reduced transit to the brain, which is already a relatively rare event—we generally do not detect brain fungal burdens within the first week of infection, even with WT cells. Free mutant cells are impaired in their ability to enter the brain (Fig. 9); it is further possible that Trojan horse transit is also reduced, due to *cdk8*’s poor intracellular survival. Together, these features of *cdk8* infection would yield delayed dissemination to the brain and a prolonged disease course where mortality is primarily determined by a relatively rare, stochastic BBB crossing event. What, however, explains the increased brain burden of the mutant? It may be that the mutant cells are less perturbing to the host, such that more are required before the mice exhibit neurological signs that trigger sacrifice. Alternatively, perhaps the slowed accumulation of fungi in the central nervous system allows the mice to tolerate higher total brain burdens.

We have shown that the Cdk8/Ssn801 system regulates the cryptococcal response to oxidative stress. Interfering with this response directly impairs the ability of the fungal cells to proliferate within macrophages, causing decreased survival in the lungs. This system also affects the virulence of C. neoformans within the brain environment, with disruption resulting in attenuation of neurological symptoms. It is possible that the variation in organ burden we see in the *cdk8* mutant strain is because the two organs offer quite different environments, each with its own selection pressures. Further studies will elucidate upstream aspects of this Kinase Module regulation of transcription in C. neoformans. Determining what environmental stimuli and cellular signaling pathways control this system will provide a better understanding of its critical role in cryptococcal adaptation to the host environment.

## MATERIALS AND METHODS

### Sequence identification and strain construction.

The cryptococcal gene encoding Cdk8 (CNAG_06086) was identified with a BLASTp analysis of the C. neoformans H99 genome, utilizing the S. cerevisiae Cdk8p amino acid sequence (YPL042C) (40). Ssn801 (CNAG_00440), Med12 (CNAG_04702), and Med13 (CNAG_03121) were identified similarly, using the S. cerevisiae Ssn8p, Med12p, and Med13p (YNL025C, YCR081W, and YDR443C, respectively) primary sequences. BLASTp analysis of the S. cerevisiae genome using the putative cryptococcal orthologs yielded the original query genes, indicating that all genes identified are reciprocal orthologs.

For strain construction, we utilized a split-marker strategy (41) to generate the prerequisite DNA for biolistic transformation (42, 43) of the C. neoformans KN99α parent strain (44). We first generated two independent *cdk8* deletion strains by replacing *CDK8* with a G418 resistance marker. We used a similar strategy to generate *ssn801*, *med12*, *med13*, and *mls1* deletion strains. To generate a *CDK8* complement strain, we replaced the G418 resistance cassette in the *cdk8* deletion strain with the native *CDK8* coding sequence followed by its native terminator and a NAT marker. We used a similar strategy to generate the *SSN801* and *MLS1* complement strains and to modify *SSN801* to express a C-terminally HA-tagged protein.

To generate the Cdk8^KD^ (kinase-dead) strain, we first identified the serine-threonine kinase domain in the Cdk8 primary structure using the MOTIF search service coupled with the PROSITE and Pfam databases; this indicated that the active motif was PROSITE entry PS00108 and the active aspartic acid residue was D225 (45). We then generated two constructs: one with the WT *CDK8* gene and one with the *CDK8* gene mutated so that the active site aspartate would instead be an inactive alanine, each in tandem with a NAT cassette. We transformed KN99α C. neoformans using the split marker strategy above and confirmed successful transformants by PCR and sequencing.

### Cell growth and growth curves.

For all studies, cryptococcal strains were grown overnight in YPD medium (1% [wt/vol] Bacto yeast extract, 2% [wt/vol] d-glucose, 2% [wt/vol] Bacto Peptone in ddH_2_O) at 30°C in room air with shaking at 230 rpm. For growth curves, cells were sedimented at 1,000 × *g* (25°C, 3 min), washed with phosphate-buffered saline (PBS), and adjusted to 1 × 10^5^ cells/ml before growth at 30°C in room air in YPD or at 37°C, 5% CO_2_ in DMEM, RPMI, or sterile artificial cerebrospinal fluid (ACSF) (124 mM NaCl, 2.5 mM KCl, 2 mM MgSO_4_, 1.25 mM KH_2_PO_4_, 26 mM NaHCO_3_, 10 mM d-glucose, 4 mM sucrose, 2.5 mM CaCl_2_, 360 mg/liter sterile BSA, 100 mM creatinine, 6 mM urea) (30). Cell counts were measured at the indicated time points, and growth curves were performed twice.

### Subcellular fractionation and immunoblotting.

Overnight cultures of desired strains were diluted to an OD_600_ of 0.5 and cultured for 3 h. Cells were sedimented by centrifugation as above and resuspended in either prewarmed YPD at 30°C or prewarmed DMEM at 37°C for 30 min. For some experiments 1.5 mM H_2_O_2_ was added to the medium. Cells were sedimented by centrifugation, washed in dithiothreitol (DTT) buffer (100 mM Tris-H_2_SO_4_, pH 9.4, 10 mM DTT), resuspended in the same, and incubated with shaking (80 rpm) for 30 min at 30°C. Cells were then washed in spheroplasting buffer (1.5 M sorbitol, 0.5% glucose, 100 mM Tris, pH 7.5, 1 mM DTT) and incubated the same way for 3 h with 10 mg/ml lysing enzyme from Trichoderma harzianum (Sigma L1412). After sedimentation, the cells were resuspended in cold homogenization buffer (0.6 M sorbitol, 10 mM Tris-HCl, pH 7.4, 1 mM EDTA, 1 mM PMSF, 0.2% [wt/vol] BSA) on ice and gently lysed with 30 strokes in a Dounce homogenizer. Cells and free nuclei were then sedimented by centrifugation of this total lysate at 1,500 × *g*, 4°C, for 15 min. The pellet was reserved (fraction 1 [see Fig. S2 in the supplemental material]), and the supernatant fraction was centrifuged at 4,000 × *g*, 4°C, for 15 min. This pellet was reserved (fraction 2), and the supernatant was further centrifuged at 15,000 × *g*, 4°C, for 30 min. The final supernatant was reserved (fraction 4), and the pellet containing mitochondria was resuspended in 5 ml of homogenization buffer (fraction 3). All samples were prepared for SDS-PAGE gel electrophoresis by 30 s of bead beating at 4°C, and for each lane 0.5% of total protein from the indicated fractions was incubated at 60°C in sample buffer (250 mM Tris-HCl, pH 6.8, 10% SDS, 30% [vol/vol] glycerol, 10 mM DTT, 0.05% [wt/vol] bromophenol blue) and resolved on a 4 to 20% gradient gel.

Protein was transferred to an activated PVDF membrane by wet transfer, and the membrane was blocked for 1 h in 5% evaporated milk in Tris-buffered saline containing Tween 20 (50 mM Tris-HCl, pH 7.4, 150 mM NaCl, 0.1% Tween 20 [TBS-T]). The membrane was washed three times in TBS-T prior to rocking overnight at 4°C with a mixture of primary antibodies in 1% milk in TBS-T. Antibodies and titers utilized were either a mixture of 1:1,000 anti-acetyl-histone H3 (Millipore Sigma 06-942) as a marker of nuclei and 1:2,000 anti-ATP5A (Abcam ab129121) as a marker of mitochondria or 1:1,000 anti-HA (Abcam ab9110) for the HA epitope alone. The membrane was washed three times in TBS-T at 25°C and then incubated with 1:10,000 secondary antibody at 25°C for 30 min (IRDye 800CW goat anti-rabbit IgG; Li-Cor 925-32211), washed three more times with TBS-T at 25°C, and immediately imaged using a Li-Cor Odyssey instrument.

### Mating assay.

Overnight cultures of desired strains were sedimented by centrifugation as above, washed with sterile PBS, and resuspended in PBS to 1 × 10^6^ cells/ml. Strains to be tested (mating type α) were then mixed 1:1 with KN99**a** cells, plated on V8 juice agar medium, pH 5.25 (46), and incubated at room temperature in the dark for 14 days. Mating filament production was imaged using a QImaging MicroPublisher 5.0 camera attached to an Olympus SZX12 dissecting microscope.

### Capsule production.

Strains were grown and washed in PBS as above, before dilution to a final density of 1 × 10^6^ cells/ml in 25 ml of prewarmed (37°C) DMEM in a 75 ml-tissue culture flask. After incubation at 37°C in 5% CO_2_ for 25 h, cells were sedimented, washed in sterile PBS, and resuspended in a 50% (vol/vol) solution of India ink. Images were taken with a Zeiss Axio Imager M2 fluorescence microscope with a Hamamatsu Flash4.0 CMOS camera.

### RNA-seq and data analysis.

RNA was isolated from experimental samples as described in references 9 and 47. Three biological replicates were tested for the WT strain and each experimental strain; each replicate was treated as an independent sample from sample preparation to sequencing. For all samples, the mean and median sequencing depth were 13.8 and 12.7 million reads, respectively. The interquartile range of sequencing depth was from 10.1 to 14.9 million reads. The mean expression of *CDK8* and *SSN801* in their respective deletion strain expression profiles was 0% and 0% of the *CDK8* and *SSN801* WT levels, confirming gene deletion.

Sequenced reads obtained from Illumina HiSeq 2500 sequencing were aligned to the C. neoformans H99 reference sequence v2 using NovoAlign version 3.07.00 with default parameters. Gene expression levels were quantified by using the reads that aligned uniquely to the reference sequence according to the HTSeq-count tool in the Python package HTSeq version 0.9.1 (48), using gene boundaries defined by version 2 of the C. neoformans genome annotation provided by the Broad Institute (49). Gene expression was normalized using the relative log expression method implemented in DESeq2 version 1.10.1 (50). Subsequently, sample quality was assessed according to the following quality control criterion on alignment statistics and gene expression profiles. Within each triplicate set for a strain, any sample for which the median of the coefficient of variation (the standard deviation divided by the mean) was above 0.2 was considered an outlier. Any outlier sample was removed from downstream analysis.

DESeq2 was used to identify genes that were differentially expressed between experimental and wild-type strains, defined as those where the absolute log_2_ fold change was greater than 0.5 and the *P* value adjusted for false-discovery rate was less than 0.005. All differentially expressed genes identified are reported in Table S1. To identify gene ontology (GO) terms that were enriched in mutant samples, each cryptococcal gene was assigned GO terms as described in reference 9. Assignments of cryptococcal genes to each of the top 10 GO terms are reported in Table S2. GO term enrichment was then analyzed using the conditional hypergeometric test implemented in GOstats version 2.36.0, R Bioconductor (51). A GO term for a given experimental strain was considered enriched if the set of DE genes within that term had a hypergeometric *P* value smaller than 0.05. All enriched GO terms are reported in Table S3. Where multiple terms within a hierarchy were enriched, we selected the most specific term possible; the top seven of this filtered set are presented in Fig. 4.

### Plate phenotyping.

Strains were grown and washed in PBS as above, before dilution to final densities of 1 × 10^7^, 1 × 10^6^, 2.15 × 10^5^, 4.65 × 10^4^, and 1 × 10^4^ cells/ml. Four microliters of each dilution was spotted onto YPD or the stress medium mentioned above and grown at 30 and 37°C. To impose cell wall stress, YPD agar (YPD medium, 2% [wt/vol] agar) was supplemented with calcofluor white at 0.050% (wt/vol), sodium dodecyl sulfate (SDS) at 0.01% (wt/vol), Congo red at 0.005% (wt/vol), and ethanol at 5% (vol/vol), or ethanol alone at 5% (vol/vol). To impose oxidative and nitrosative stress, YNB (0.67% [wt/vol] yeast nitrogen base without amino acids [Difco, 291940], 2% [wt/vol] agar, 2% [wt/vol] d-glucose, 25 mm sodium succinate) was supplemented with either 0.5 mM H_2_O_2_ or 0.5 mM NaNO_2_, with or without 1 M sorbitol. To test melanin production, 5 μl of cells at 5 × 10^6^ cells/ml was spotted onto YNB agar plates supplemented with 1 mg/ml d-glucose, 1 mg/ml l-glycine, 4 mg/ml KH_2_PO_4_, 0.46 mg/ml MgSO_4_·7H_2_O, 0.5 μg/ml d-biotin, 0.5 μg/ml thiamine, and 0.2 mg/ml l-3,4-dihyroxyphenylalanine (l-DOPA) (52, 53). Melanization plates were incubated for 3 days at 30 and 37°C.

### Mitochondrial morphology.

Strains were grown and washed in PBS as above, diluted to a final density of 1 × 10^6^ cells/ml in 25 ml of prewarmed (37°C) DMEM in a 75 ml-tissue culture flask, and incubated at 37°C, 5% CO_2_ for 24 h. Cells were then harvested as above, washed with PBS, and resuspended in 1 ml PBS with 1 mM MitoTracker CMXRos. After 1 h of incubation, the cells were washed three times in sterile PBS and imaged with a Zeiss Axio Imager M2 fluorescence microscope with a Hamamatsu Flash4.0 CMOS camera. A minimum of 100 cells per sample were categorized as having fragmented, tubular, or diffuse morphology. All samples were analyzed in a blinded fashion; experiments were independently performed three times and analyzed using Fisher’s exact test of independence (54).

### Intracellular survival assays.

THP-1 cells (1 ml of 1.67 × 10^5^ cells/ml in 24-well tissue culture plates) were differentiated in THP-1 medium (RPMI supplemented with 10% heat-inactivated fetal bovine serum [FBS], 48 µM β-mercaptoethanol, 1 mM sodium pyruvate, 100 μM penicillin, 100 U/ml streptomycin) by incubating for 48 h at 37°C in 5% CO_2_ in the presence of 25 nM phorbol 12-myristate 13-acetate (PMA). They were then permitted to recover for 24 h in THP-1 medium without PMA (55). In parallel, cryptococcal strains were grown overnight in YPD and washed as above, opsonized in 40% human serum in PBS (30 min at 37°C at 1 × 10^7^ cells/ml), washed in PBS, and resuspended in prewarmed (37°C) RPMI. Opsonized fungi were added to differentiated THP-1 cells at an MOI of 0.1 in triplicate wells in three parallel plates (26, 56). The plates were incubated for 1 h to permit uptake of opsonized cells, washed twice with sterile prewarmed PBS, and refilled with prewarmed THP-1 medium. They were then incubated for 0, 24, or 48 h at 37°C, 5% CO_2_ before being washed twice with sterile PBS, refilled with sterile ddH_2_O, and incubated at room temperature for 30 min to lyse macrophages before plating on YPD agar to quantify CFU. In some assays, reactive oxygen species scavengers (8 μg/ml bovine erythrocyte superoxide dismutase [Sigma S5395], 80 μg/ml bovine liver catalase [MP Biomedicals, 02100429], and 100 mM d-mannitol [Sigma M1902]) were added at 6-h intervals beginning at 0 h. In all studies, fold changes in CFU over time were compared using one-way ANOVA with Dunnett’s multiple-comparison *post hoc* test. This normalizes for any difference in phagocytosis.

Bone marrow-derived macrophages (BMDM) were obtained by isolating bone marrow from C57BL/6 mouse (Jackson Laboratory) femurs and expanding the cellular population by growth in BMDM medium (RPMI supplemented with 20% heat-inactivated FBS, 33% L cell supernatant, 100 μM penicillin, 100 U/ml streptomycin) for 7 days. Macrophages were then isolated using anti-F4/80 conjugated biotin (Invitrogen, 13-4801-82) coupled with anti-biotin magnetic microbeads (Miltenyi Biotec, 130-090-485) loaded in a MACS separation column (Miltenyi Biotec, 130-042-201) after blocking with FC block (BD Biosciences, 553142). Cells were plated at the same density as THP-1 cells, and intracellular survival of fungi was assayed as above.

### Virulence and ethics statement.

Overnight cultures of the desired strains were harvested by centrifugation at 1,000 × *g*, washed with sterile PBS, and diluted to 2.5 × 10^5^ cells/ml in sterile PBS. For long-term mouse studies, groups of six 4- to 6-week-old female BALB/c (Jackson Laboratory) mice were anesthetized by injection with 0.24 mg xylazine and 1.20 mg ketamine in 120 μl sterile water and intranasally inoculated with 1.25 × 10^4^ cryptococcal cells. Groups of three mice from WT- and *cdk8* strain-infected groups were sacrificed at days 6 and 12 to monitor infection; these mice were not included in the survival curve. The remaining mice were monitored and humanely sacrificed in a carbon dioxide chamber whenever their weights decreased to below 80% of their maximum measured weight. For all mice, lung and brain homogenates were prepared and plated on YPD agar to quantify CFU. Organ burdens were analyzed by one-way analysis of variance (ANOVA) with Dunnett’s multiple-comparison *post hoc* test. All animal protocols were approved by the Washington University Institutional Animal Care and Use Committee (reference 20170131), and care was taken to minimize handling and discomfort to the animals.

### Transwell blood-brain barrier crossing.

Model blood-brain barriers were generated from a brain microvascular endothelial cell line in 24-well transwell plates, and crossing was assayed at 20 h exactly as described in reference 4. Triplicate experiments were performed, and results were analyzed by one-way ANOVA with Dunnett’s multiple-comparison *post hoc* test.

### Data availability.

All data are available without restriction. RNA-seq data are available at the NCBI GEO database (accession number GSE125281).
