# Supplementary material for: Cdk8 and Ssn801 Regulate Oxidative Stress Resistance and Virulence in Cryptococcus neoformans
Source: mBio. 2019 Feb 12;10(1):e02818-18. doi: 10.1128/mBio.02818-18 (PMC6372802; doi:10.1128/mBio.02818-18)
Supplement: FIG S1 [file mBio.02818-18-sf001.pdf]

FIGURE S1

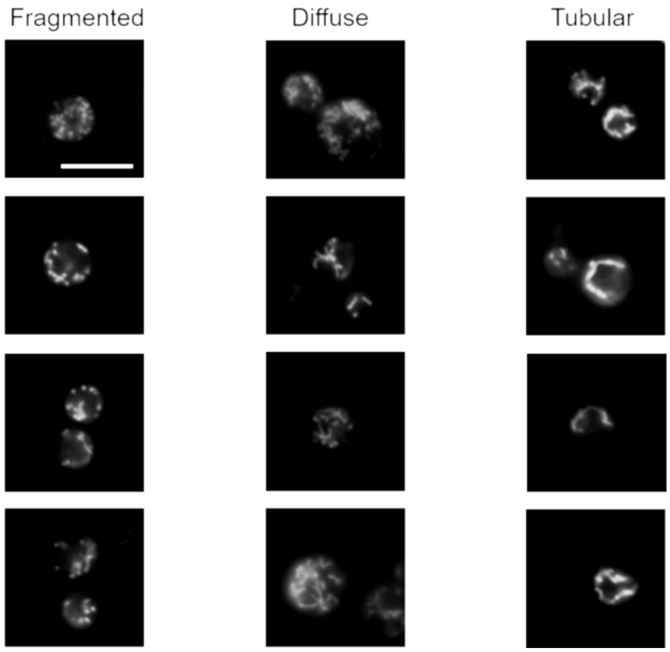

Fig S1: Additional examples of categories of mitochondrial morphology.

Wild type cells were grown and mitochondrial morphology assessed as in the Methods. Example micrographs of the different categories of mitochondrial morphology are shown. Scale bar, 10 μm. The top and bottom panels in the Diffuse column are shown at slightly lower brightness for clarity and the second panel in the Tubular column shows a larger cell exhibiting tubular morphology together with a smaller cell of fragmented morphology.
