# Supplementary material for: Cdk8 and Ssn801 Regulate Oxidative Stress Resistance and Virulence in Cryptococcus neoformans
Source: mBio. 2019 Feb 12;10(1):e02818-18. doi: 10.1128/mBio.02818-18 (PMC6372802; doi:10.1128/mBio.02818-18)
Supplement: FIG S2 [file mBio.02818-18-sf002.pdf]

FIGURE S2

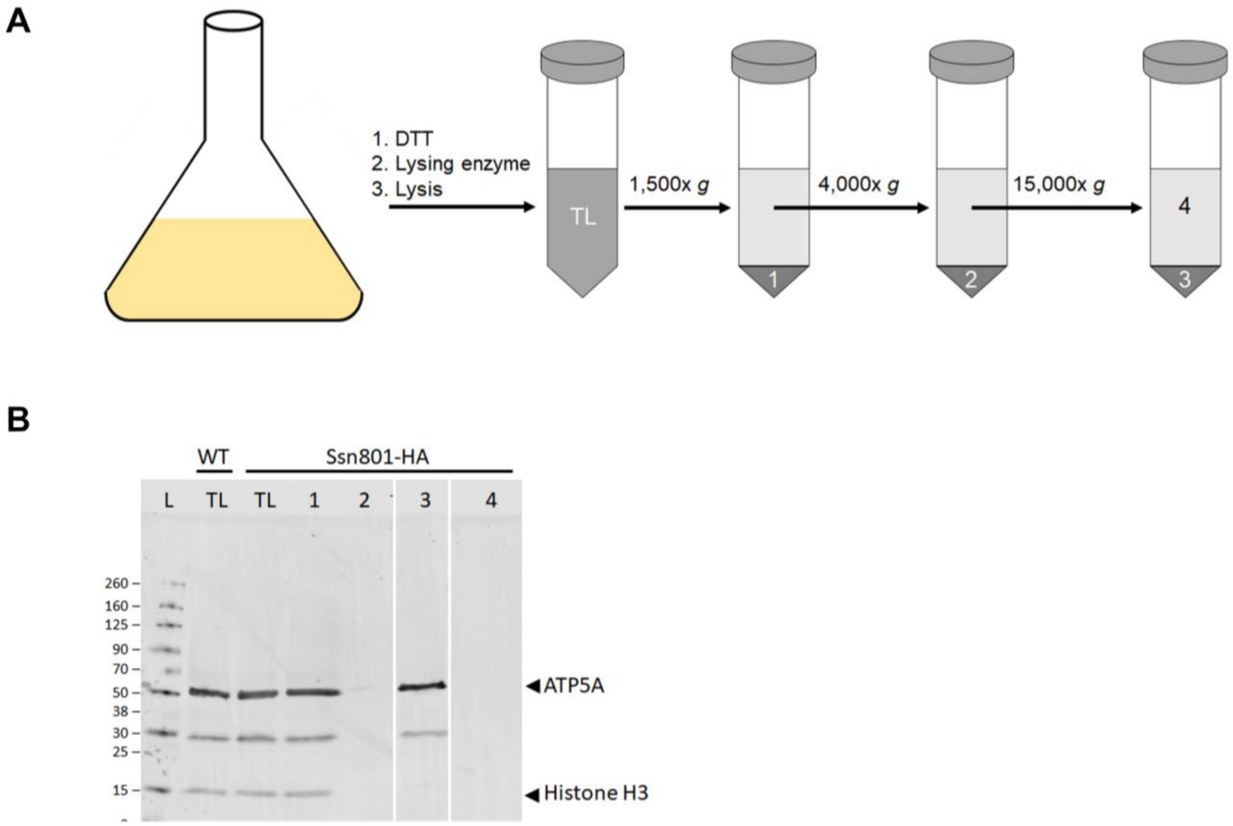

Fig S2: Subcellular fractionation of *C. neoformans*.

(A) Schematic of isolation of mitochondria by subcellular fractionation; see Methods for details. (B) Immunoblot of fractions indicated in A. Nuclei, marked by histone H3 (16 kDa), are present only in the total lysate (TL) and 1,500x *g* pellet (1). In contrast mitochondria, marked by ATP5A (55 kDa), are present in the TL, 1,500x *g* pellet (1; in unbroken cells), and the 15,000x *g* pellet (3). The band at ~29 kDa is a consistent background band from the anti-ATP5A antibody. L, ladder.
