# Supplementary material for: Cdk8 and Ssn801 Regulate Oxidative Stress Resistance and Virulence in Cryptococcus neoformans
Source: mBio. 2019 Feb 12;10(1):e02818-18. doi: 10.1128/mBio.02818-18 (PMC6372802; doi:10.1128/mBio.02818-18)
Supplement: FIG S3 [file mBio.02818-18-sf003.pdf]

FIGURE S3

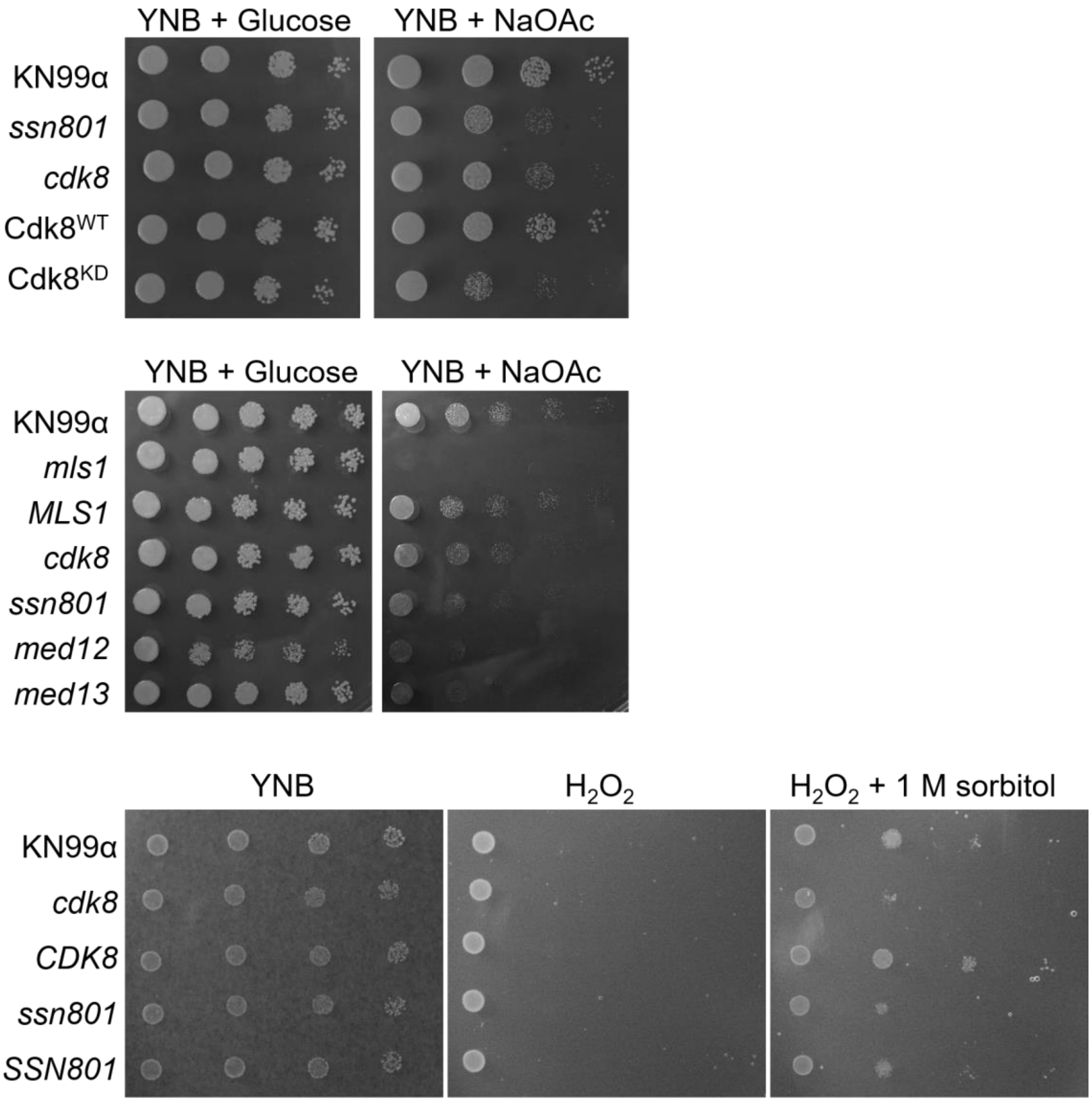

Fig S3: Growth of mutant strains on acetate and under oxidative stress with sorbitol

Serial 10-fold dilutions (10^7 to 10^4 cells/mL) of the strains listed at the left were tested for glyoxylate cycle function by growth on YNB with glucose or acetate to (top two panels) or were tested for growth under peroxide stress with or without sorbitol (bottom panel).
