# Supplementary material for: Cdk8 and Ssn801 Regulate Oxidative Stress Resistance and Virulence in Cryptococcus neoformans
Source: mBio. 2019 Feb 12;10(1):e02818-18. doi: 10.1128/mBio.02818-18 (PMC6372802; doi:10.1128/mBio.02818-18)
Supplement: FIG S4 [file mBio.02818-18-sf004.pdf]

FIGURE S4

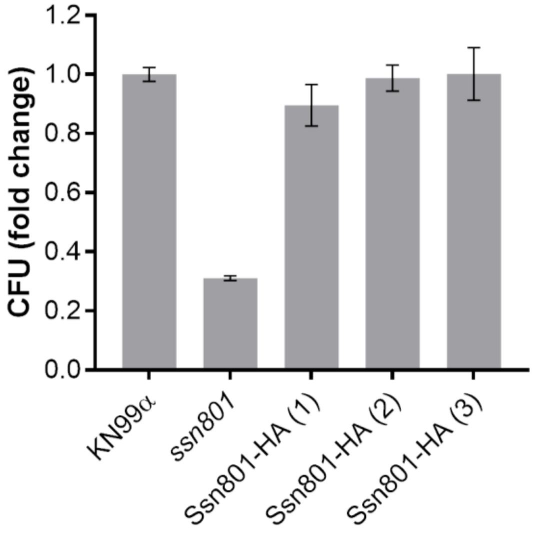

Fig S4: Ssn801-HA strains show WT levels of survival in THP-1 macrophages compared to the *ssn801* mutant strain.

Intracellular survival (mean ± SD) of the indicated strains is shown as fold change, normalized to WT (1.8-fold change). Results shown are representative of three independent experiments. $p \leq 0.0001$ for *ssn801* compared to WT.
