# Supplementary material for: Cdk8 and Ssn801 Regulate Oxidative Stress Resistance and Virulence in Cryptococcus neoformans
Source: mBio. 2019 Feb 12;10(1):e02818-18. doi: 10.1128/mBio.02818-18 (PMC6372802; doi:10.1128/mBio.02818-18)
Supplement: FIG S5 [file mBio.02818-18-sf005.pdf]

FIGURE S5

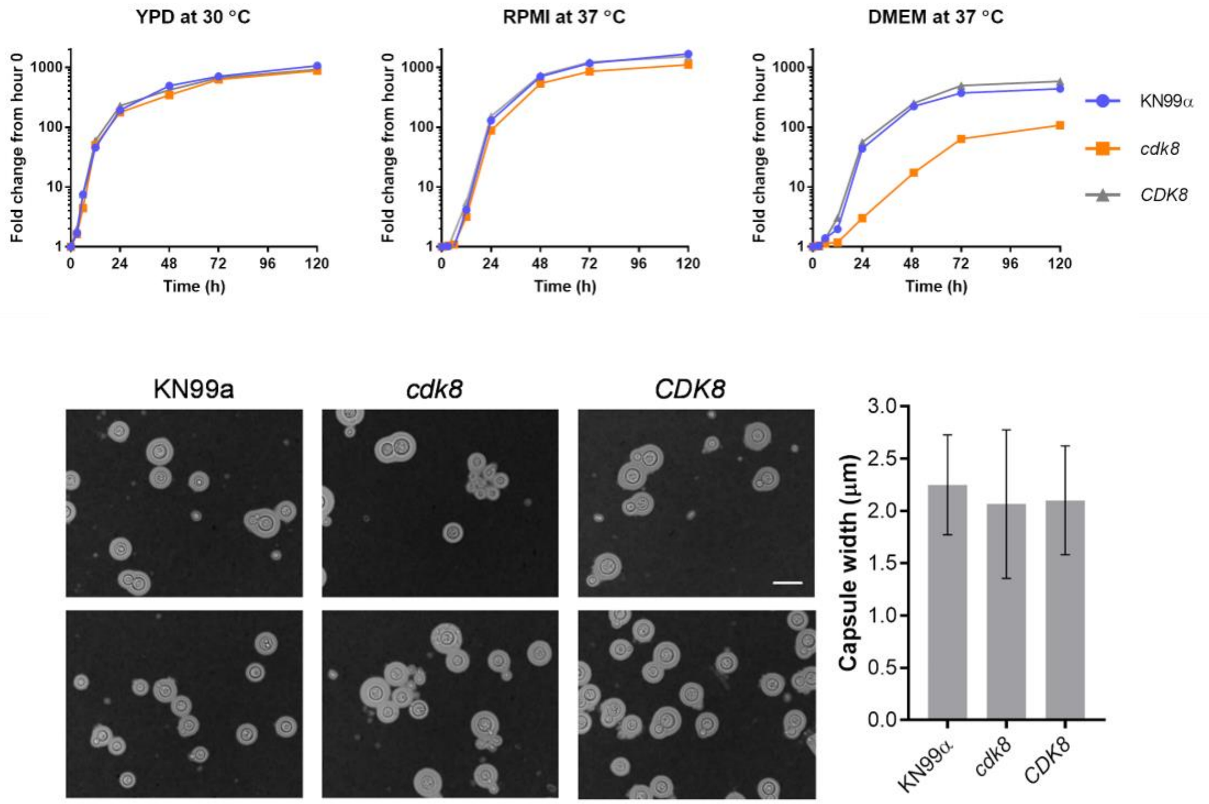

Fig S5: ***cdk8*** **cells grow like WT in YPD and RPMI (although not DMEM) and exhibit normal capsule.**

Top row, growth curves (mean ± SD) of WT (KN99α, blue), *cdk8* (orange), and the complemented mutant strain (*CDK8*, gray) in (A) YPD, (B) RPMI, and (C) DMEM. RPMI medium was used in the studies reported in the intracellular survival studies in the main text. Bottom left, example micrographs of the indicated strains after 24 h of incubation in capsule-inducing conditions (DMEM, 37 °C, 5% CO_2) and India ink staining. All images are to the same scale; scale bar, 10 µm. Bottom right, capsule width measured for at least 100 cells chosen at random. Mean ± SD is plotted. $p \geq 0.05$ for *cdk8* compared to WT.
