# Supplementary material for: Cdk8 and Ssn801 Regulate Oxidative Stress Resistance and Virulence in Cryptococcus neoformans
Source: mBio. 2019 Feb 12;10(1):e02818-18. doi: 10.1128/mBio.02818-18 (PMC6372802; doi:10.1128/mBio.02818-18)
Supplement: FIG S6 [file mBio.02818-18-sf006.pdf]

FIGURE S6

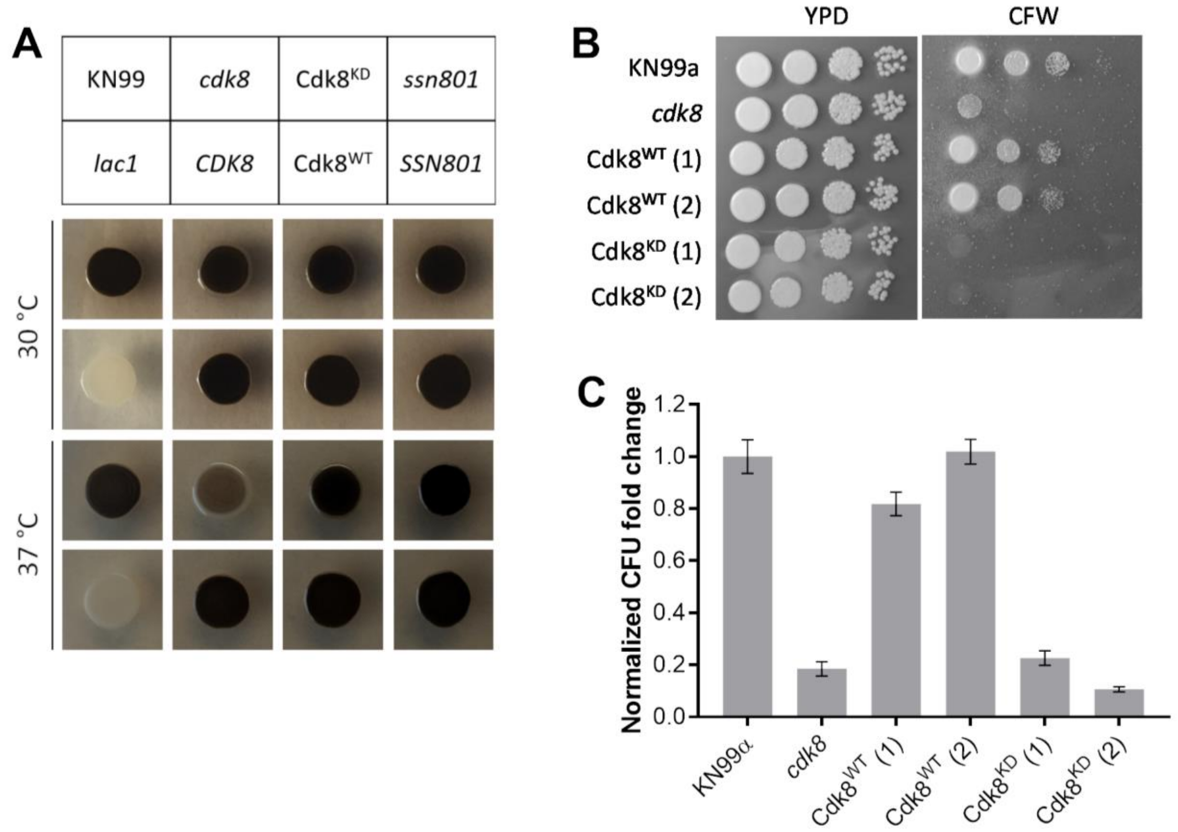

Fig S6: Cdk8 kinase activity is not required for melanin production but is required for survival on a cell wall stressor.

(A) Melanization of Kinase Module mutants. The indicated strains (each 10^4 cells) were grown at 30 and 37 °C on L-DOPA agar to assess melanin production (see Methods for details). Cdk8 kinase activity is required for robust growth in the presence of (B) a cell wall stressor and (C) THP-1 macrophages. Serial 10-fold dilutions (10^7 to 10^4 cells/mL) of the strains listed at the left were grown on the medium indicated.
