# Supplementary material for: Cdk8 and Ssn801 Regulate Oxidative Stress Resistance and Virulence in Cryptococcus neoformans
Source: mBio. 2019 Feb 12;10(1):e02818-18. doi: 10.1128/mBio.02818-18 (PMC6372802; doi:10.1128/mBio.02818-18)
Supplement: FIG S7 [file mBio.02818-18-sf007.pdf]

FIGURE S7

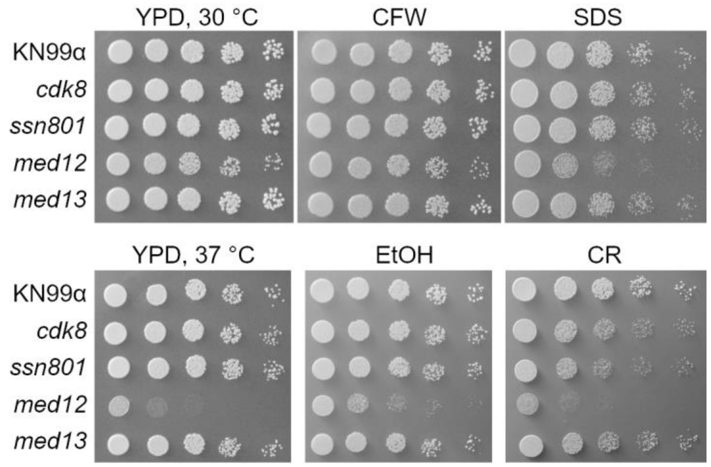

Fig S7: Cells lacking Med12, but not Med13, are defective in growth at 37 °C and under some stress conditions.

The strains indicated were plated on YPD medium in the conditions shown. CFW, 0.05% w/v calcofluor white at 37 °C; SDS, 0.01% w/v sodium dodecyl sulfate at 30 °C; 5% v/v ethanol at 30 °C; CR, 0.005% Congo red and 5% v/v ethanol at 30 °C.
